# The Functional DRD3 Ser9Gly Polymorphism (rs6280) Is Pleiotropic, Affecting Reward as Well as Movement

**DOI:** 10.1371/journal.pone.0054108

**Published:** 2013-01-24

**Authors:** Jonathan Savitz, Colin A. Hodgkinson, Chantal Martin-Soelch, Pei-Hong Shen, Joanna Szczepanik, Allison Nugent, Peter Herscovitch, Anthony A. Grace, David Goldman, Wayne C. Drevets

**Affiliations:** 1 Laureate Institute for Brain Research, and Department of Psychiatry, University of Oklahoma College of Medicine, Tulsa, Oklahoma, United States of America; 2 Department of Medicine, Tulsa School of Community Medicine, Tulsa, Oklahoma, United States of America; 3 Section on Neuroimaging in Mood and Anxiety Disorders, Mood and Anxiety Disorders Program, NIH/NIMH, Bethesda, Maryland, United States of America; 4 Laboratory of Neurogenetics, National Institute on Alcohol Abuse and Alcoholism, NIH, Rockville, Maryland, United States of America; 5 Department of Psychiatry and Psychotherapy, University Hospital Zurich, Zurich, Switzerland; 6 Experimental Therapeutics, Mood and Anxiety Disorders Program, NIH/NIMH, Bethesda, Maryland, United States of America; 7 Clinical Center, National Institutes of Health, Bethesda, Maryland, United States of America; 8 Departments of Neuroscience, Psychiatry and Psychology, University of Pittsburgh, Pittsburgh, Pennsylvania, United States of America; 9 Department of Psychiatry, University of Oklahoma College of Medicine, Tulsa, Oklahoma, United States of America; University of Wuerzburg, Germany

## Abstract

Abnormalities of motivation and behavior in the context of reward are a fundamental component of addiction and mood disorders. Here we test the effect of a functional missense mutation in the dopamine 3 receptor (DRD3) gene (ser9gly, rs6280) on reward-associated dopamine (DA) release in the striatum. Twenty-six healthy controls (HCs) and 10 unmedicated subjects with major depressive disorder (MDD) completed two positron emission tomography (PET) scans with [^11^C]raclopride using the bolus plus constant infusion method. On one occasion subjects completed a sensorimotor task (control condition) and on another occasion subjects completed a gambling task (reward condition). A linear regression analysis controlling for age, sex, diagnosis, and self-reported anhedonia indicated that during receipt of unpredictable monetary reward the glycine allele was associated with a greater reduction in D2/3 receptor binding (i.e., increased reward-related DA release) in the middle (anterior) caudate (p<0.01) and the ventral striatum (p<0.05). The possible functional effect of the ser9gly polymorphism on DA release is consistent with previous work demonstrating that the glycine allele yields D3 autoreceptors that have a higher affinity for DA and display more robust intracellular signaling. Preclinical evidence indicates that chronic stress and aversive stimulation induce activation of the DA system, raising the possibility that the glycine allele, by virtue of its facilitatory effect on striatal DA release, increases susceptibility to hyperdopaminergic responses that have previously been associated with stress, addiction, and psychosis.

## Introduction

**Figure 1:**
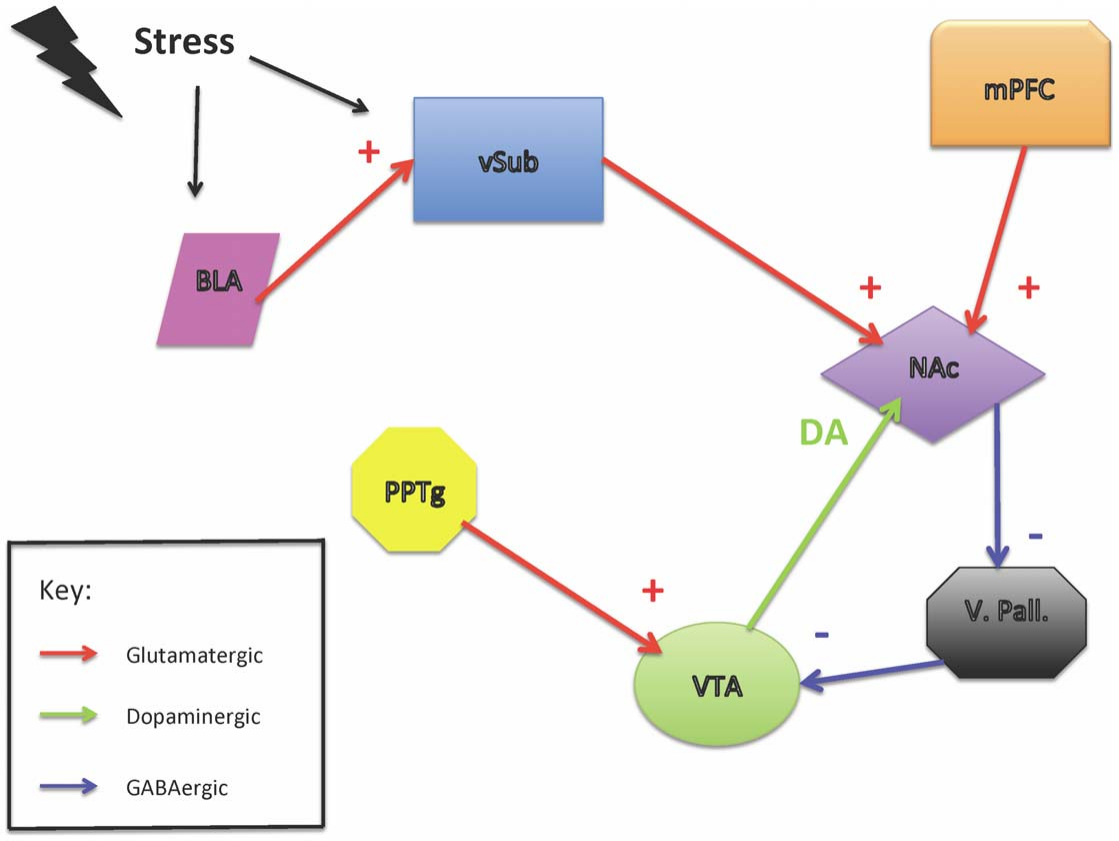
The phasic burst-firing activity of DA neurons in the VTA is induced by direct excitatory input from the PPTg to the VTA. (Adapted from Grace et al. 2007). Tonic firing (population firing) is regulated by a loop consisting of the vSub, NAc, VP, and VTA. Activation of the vSub, either directly or via the BLA drives NAc-mediated disinhibition of the VTA, resulting in tonic DA release. Burst-firing can only occur in the proportion of the DA neuron population that is tonically active prior to the arrival of afferent excitatory transmission from the mPFC or the PPTg. BLA = basolateral amygdala, mPFC = medial prefrontal cortex, NAc = nucleus accumbens, PPTg = peduculopontine tegmentum, VP = ventral pallidum, vSub = ventral subiculum, VTA = ventral tegmental area. Red arrows = glutamatergic projections, blue arrows = GABAergic projections, green arrow = dopaminergic projections.

Abnormalities of motivation and behavior in the context of reward are a key component of addiction and mood disorders. While the neurophysiological structure of the “reward circuit” has been well delineated (figure 1), the genetic contribution to striatal dopaminergic signaling remains poorly understood. Using positron emission tomography (PET), a number of groups have reported associations between functional variants in the DRD2 gene and the binding potential (BP) of [^11^C]raclopride, a D2/3 antagonist [1], [2], [3], [4]. The BP parameter reflects the density x the affinity of D2/3 receptors in the brain. These imaging data are potentially important because genetically-driven differences in dopamine (DA) receptor function may influence the changes in dopaminergic signaling that modulate emotional, motivational and stress responses.

In contrast, the impact of genetic variation in the DRD3 gene on measures of DA receptor binding or function has received little attention in the PET literature. Unlike D1 and D2 receptors, the D3 receptor is largely expressed on the dopaminergic neurons of the nucleus accumbens (NAc) [5] where it acts as a DA release-regulating autoreceptor (the D3 receptor has also been shown to act as a heteroreceptor) [6]. Other regions in which the D3 receptor is expressed include the substantia nigra, hypothalamus, globus pallidus, and thalamus [7], [8]. In preclinical studies, blockade of the D3 receptor has been reported to reduce relapse after the presentation of conditioned alcohol and nicotine-associated cues [9], [10] and to prevent the stress-induced reinstatement of cocaine-seeking behavior [11]. In humans, *postmortem* work showed that the density of D3 receptors was elevated in cocaine abusers [12] while polymorphisms of DRD3 gene have been associated with smoking behavior [13], [14], nicotine dependence [15], and alcohol craving [16]. Polymorphisms of the DRD3 gene also have been implicated in increasing the risk for major depressive disorder (MDD) [17], [18].

Stress-induced anhedonia, the failure to experience pleasure in appropriate contexts, has been associated with decreased responsiveness of the D3 receptor in the NAc in animal models; conversely multiple classes of antidepressant medications as well as electroconvulsive therapy (ECT) enhance D3 receptor responsiveness [19], [20], [21].. Consistent with these data, genetic variants in the DRD3 gene were associated with differential responsiveness to ECT in humans with major depressive disorder (MDD) [22]. Notably, pramipexole, a dopamine receptor agonist with high selectivity for the D2 dopamine receptor family and preferential affinity for the D3 receptor subtype, has shown antidepressant effects in humans [23], [24] and has been reported to augment tonic DA release and to alter DA neuronal firing activity in rodents [25].

Pharmacogenetic studies have reported that a functional single nucleotide polymorphism (SNP) in the DRD3 gene (ser9gly) influenced antidepressant response in bipolar disorder patients treated with a combination of olanzapine and fluoxetine [26] as well as response to paroxetine in patients with MDD [27]. The ser9gly polymorphism has also been associated with depression arising in the context of dementia [28] as well as primary MDD [17], [29], [30].

A potentially informative measure of the effect of DRD3 polymorphisms on central dopaminergic function is afforded by the sensitivity of [^11^C]raclopride binding to endogenous DA release [31], given the prominent role of the D3 receptor in regulating DA release in the ventral striatum [6]. This PET-[^11^C]raclopride technique thus allows exploration of the effects of genetic variation on the amount of DA released under conditions associated with increased phasic release of DA, such as during receipt of unpredicted reward [32]. Since DA is released in the NAc in response to unpredicted reward [33], the degree of displacement of [^11^C]raclopride by the increase in intrasynaptic DA concentrations during reward would then be evident by changes in the radioligand binding to either D2 or D3 receptors [34], [35].

Notably, the application of the PET-[^11^C]raclopride imaging to measure the degree of DA release during reward provides an experimental paradigm that is sensitive to potential functional effects of genetic variation in the D3 receptor, despite the fact that at baseline, the [^11^C]raclopride signal is dominated by the D2 receptor. [^11^C]Raclopride shows not only a modestly greater affinity for the D2 receptor compared with the D3 receptor [36], but relative to the D3 receptor, the density of the D2 receptor is approximately 2-fold greater in the NAc and 4-fold greater in the caudate at *postmortem* [37]. More recent *in vivo* studies using [^11^C]-(+)-PHNO, a radioligand that binds preferentially to the D3 receptor, have indicated that in the absence of reward, the D3 receptor accounts for less than 10% of the signal in the striatum and caudate [7], [8]. Thus the D3 receptor binding contributes only a fraction of the baseline [^11^C]raclopride BP rendering this measure relatively insensitive to genetically-mediated differences in D3 receptor binding (which would likely be masked by noise introduced by variations in D2 receptor phenotype).

In healthy controls (HC), we previously assessed the magnitude of reward-associated DA release with PET by measuring differences in D2/D3 receptor BP_ND_ (ΔBP_ND_) for [^11^C]raclopride between an unpredictable monetary reward condition and a sensorimotor control condition [38]. A decrease in BP_ND_, indicative of increased endogenous DA release, was observed in the ventral striatum and left middle caudate region-of-interest (anterior caudate head) regions-of-interest in the reward versus the control condition [38]. We also found a significant reduction in [^11^C]raclopride BP_ND_ in depressed patients versus HCs in the anteroventral striatum (unpublished data). Here we tested the effect of a functional missense mutation in the DRD3 gene (ser9gly, rs6280) on ΔBP_ND_ in the caudate and striatum in healthy individuals and MDD patients who completed PET scanning with [^11^C]raclopride.

## Patients and Methods

### Subject Data

Subjects provided written informed consent after receiving a full explanation of the study procedures and risks, as approved by the NIMH IRB. Twenty-six right-handed HCs (15 women; mean age 34.4±8.3) and 10 currently depressed, unmedicated patients with MDD (8 women; mean age 38.2±11.3) participated in the study (table 1). Handedness was assessed using the Edinburgh Handedness Inventory. A medical history, physical examination, laboratory testing (including drug screening), T1-weighted MRI, and both structured (Structured Clinical Interview for the DSM-IV-TR) and an unstructured (with a psychiatrist) psychiatric interviews were obtained on all participants. Anhedonic symptoms were rated using the Snaith-Hamilton Pleasure Scale (SHAPS).

**Table 1:** Demographic and Clinical Data for MDD and HC Groups (A) and Total Sample Stratified by Genotype (B).

*A*	*MDD*	*HC*
N	10	26
Gender (M/F)	2/8	11/15
Age	38.2±11.3	34.5±8.3
MADRS	24.3±8.2	0.6±1.6
HAM-A	12.9±6.6	0.7±1.6
Chapman-P	20.0±10.5	8.0±5.1
Chapman-S	18.2±9.2	5.6±3.9
SHAPS	32.3±7.7	20.1±6.5

*B*	*SER/SER*	*SER/GLY*	*GLY/GLY*
N	13	11	12
Diagnosis (MDD/HC)	4/9	4/7	2/10
Gender (M/F)	4/9	5/6	4/8
Age	35.2±9.5	39.0±11.0	32.6±6.3
Baseline BP_ND_: VS	4.24±1.00	4.43±1.01	3.85±1.21
Baseline BP_ND_: MC	3.77±0.94	3.75±1.07	3.56±1.11
Δ BP_ND_: VS	0.45±7.34	−2.15±6.73	−6.46±8.07
Δ BP_ND_: MC	2.67±7.79	−2.09±5.90	−5.70±5.10

Note: Δ BP_ND_ = Change in Raclopride Binding Between Baseline Sensorimotor and Gambling Task; Chapman-P = Chapman Physical Anhedonia Rating Scale; Chapman-S = Chapman Social Anhedonia Rating Scale; F = Female; HAM-A = Hamilton Anxiety Scale; HC = Healthy Control; M = Male; MADRS = Montgomery-Asberg Depression Rating Scale; MC = Middle Caudate; VS = Ventral Striatum; MDD = Major Depressive Disorder; SHAPS = Snaith-Hamilton Pleasure Scale.

Volunteers were excluded if they had taken antidepressant medications or other drugs likely to affect cerebral physiology, monoaminergic neurotransmitter function, or vascular function within 3 weeks of the scan (8 weeks for fluoxetine). Other exclusion criteria included having: a major medical or neurological disorder; a history of psychosis; illicit drug use or alcohol abuse within one year of the scan; lifetime history of alcohol or drug dependence; lifetime exposure to stimulant drugs (e.g., cocaine, amphetamines) tobacco use within 1 year of the scan; lifetime history of pathological gambling behavior as assessed with the lie/bet screen and South Oaks Gambling Screen (SOGS), electrolyte disturbance, anemia, positive drug test or HIV screen on laboratory testing, IQ<85; current pregnancy or breast feeding, and general MRI exclusions. Additional exclusions applied to the healthy group included having met DSM-IV-TR criteria for a current or past psychiatric disorder or having a first degree relative with a mood or anxiety disorder as established using the Family Interview for Genetic Studies (FIGS).

### PET Imaging

#### The Sensorimotor and Gambling (Slot Machine) Tasks

Methodological details can be found in [38]. Briefly, the slot machine task involved two conditions, each consisting of 180 trials, each of average duration 8 sec. In the monetary-reward condition subjects received financial rewards unpredictably, in a pseudo-randomized order with an average of one reward per four trials. In the sensorimotor control condition subjects performed the same task without receiving rewards, to control for motor activation or other nonspecific aspects of task-performance (figure S1).

During scanning subjects initially rested for 20 min in order to allow [^11^C]raclopride to approach equilibrium (previously validated by [39], [40]), and thereafter they performed the sensorimotor control task for approximately 24 min. Beginning 50 min after the start of the [^11^C]raclopride infusion, subjects performed the monetary reward condition for approximately 24 min. This timing allowed the distribution of [^11^C]raclopride to come into equilibrium for each of the two task conditions. During the 24 min epochs that corresponded to each task condition, subjects alternated between two-min periods in which they actively performed the task and one-min periods when they rested to minimize fatigue. Each subject won a total of $33 during the rewarded condition.

#### Image Acquisition

PET scans were acquired using a GE-Advance scanner in 3D-mode (3D resolution = 6 mm full-width at half-maximum). The data were reconstructed using a Hanning-filter and Gaussian-fit scatter-correction method. A transmission scan was acquired using rotating 68Ge/68Ga rods to perform attenuation-correction of the emission scans. The [^11^C]raclopride (20 mCi) was administered as an initial bolus over 60 seconds followed by a maintenance infusion over the remainder of the scanning session using a computer-controlled pump. Dynamic emission scanning (27 frames) was initiated with injection of the [^11^C]raclopride bolus. MRI images were acquired on a General Electric 3.0 Tesla scanner (GE Healthcare, Waukesha, WI), using T1 weighted sequences to provide an anatomical framework for image analysis (in-plane resolution: 0.86 mm, slice thickness: 1.2 mm).

#### PET Image Analysis

Mean tissue radioactivity concentrations from the baseline and reward images were extracted using MRI-based regions-of-interest (ROI), defined on a template MRI image using MEDx software in the anteroventral striatum, middle caudate, and cerebellum after Martin-Soelch et al. (2011). Each individual's MRI was registered to a template brain and the ROIs were repositioned as needed to accommodate individual differences in anatomy. The anatomical accuracy of each set of individual ROI was verified by a neuroscientist familiar with striatal anatomy (WCD). These ROIs then were back-transformed into the subject's native MRI space and applied to the co-registered PET images.

Decay-corrected, tissue radioactivity concentrations (C) were obtained from each ROI using a calibrated phantom standard to convert tomographic counts to Bq/ml. Mean radioactivity in the reference region (cerebellum; C′) was used to factor out the effects of free and non-specifically bound [^11^C]raclopride. The percent change in [^11^C]raclopride binding was computed as the difference in BP_ND_ (C/C′-1 for each ROI) between baseline and reward images.

#### Selection of Regions of Interest

The ventral striatum and middle caudate were chosen as ROIs on the basis of our previously published imaging findings regarding the striatal subregions where [^11^C]raclopride binding changed in response to unpredicted monetary reward [38]. The ROIs in [38] were defined using neuroanatomical landmarks on the co-registered MRI scan after the method described by [41]. In primates the cells with histochemical and connectional features of the NAc shell implicated in reward learning are scattered through the accumbens area, ventromedial caudate, and anteroventral putamen [42], and previous PET studies of amphetamine-induced DA release showed that euphoria correlated with DA release in the anteroventral striatum and ventral putamen [41], [43]. The middle caudate consists of the central portion of the anterior caudate head, which receives anatomical projections from the orbitofrontal and ventromedial prefrontal cortical regions implicated in reward processing [44].

### Genotyping

Samples were genotyped for the rs6280 polymorphism using the Illumina GoldenGate platform [45]. The array covers 130 candidate genes for alcoholism, other addictions, and mood and anxiety disorders. The average call rate of this platform is 98.3% and the replication rate, 99.7%. The array also allows for control of population stratification through the inclusion of 186 ancestry informative markers (AIMs). The AIMs panel identifies six distinct groups from the global population, based upon geographic relationships: Europe, Africa, Middle East, Asia, Far East Asia, Oceania, and the Americas.

### Statistical Analysis

To test whether ethnicity predicted D3 [^11^C]raclopride binding, we used African and European ancestry, defined by genetically informative markers, as predictors of [^11^C]raclopride binding in separate regression models. There was no effect of either African or European ancestry on baseline BP_ND_ of ventral striatum and middle caudate as well as Δ BP_ND_ of the ventral striatum and middle caudate (table S1). The majority of the participants were Caucasian or African American and thus the ancestry categories, Middle-Eastern, Asian, Far East Asian, Oceania, and America were not evaluated as predictors of raclopride binding because these factors were quantitatively negligible across the sample.

Imaging genetics data were analyzed with a 2-step forced linear regression. The following predictor variables were entered into the model: age, sex, diagnosis, and SHAPS score (step 1) and ser9gly genotype (ser/ser vs ser/gly vs gly/gly) (step 2). Baseline BP_ND_ of ventral striatum and middle caudate as well as Δ BP_ND_ of the ventral striatum and middle caudate constituted the dependent variables.

Hardy-Weinberg Equilibrium (HWE) was tested using Haploview [46]. Thirteen subjects were homozygous for the serine allele, 11 subjects were heterozygotes, and 12 subjects were homozygous for glycine.

## Results

**Figure 2:**
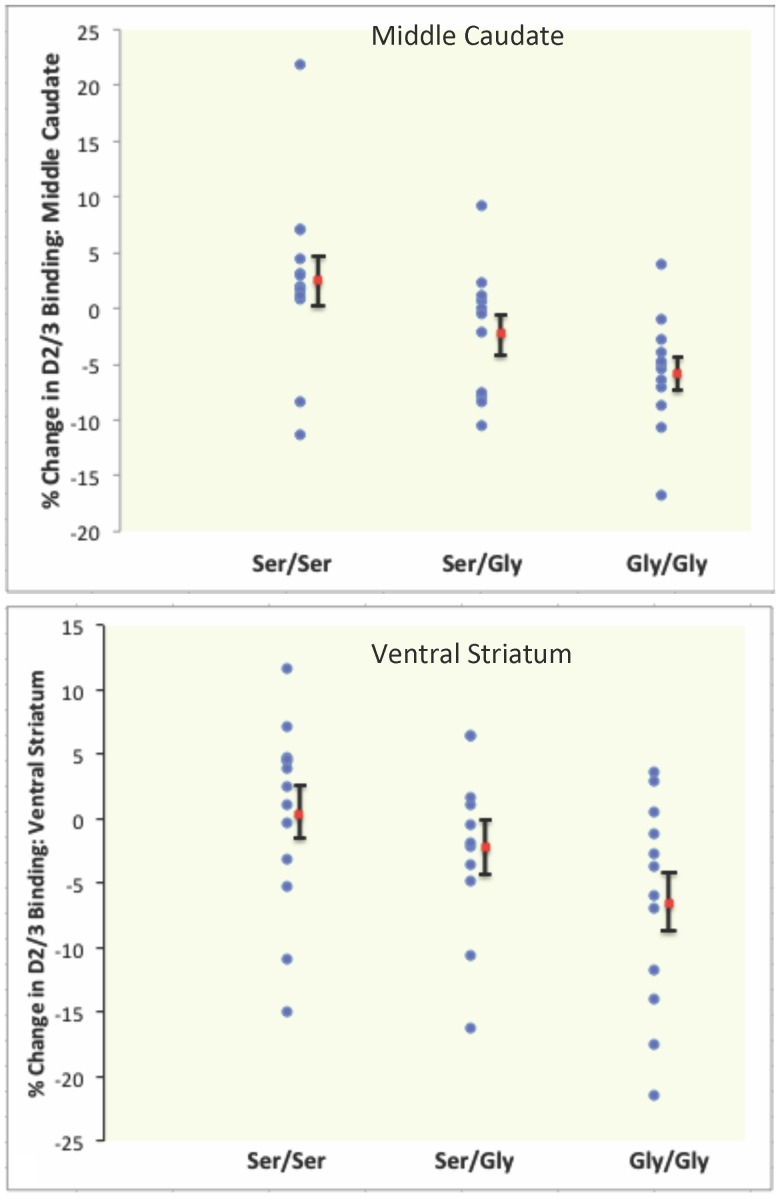
Scatterplots of the percent change in D2/D3 receptor binding in the reward versus baseline condition for the middle caudate and the ventral striatum. The mean change in D2/D3 receptor binding for each group is represented by the red square and the standard error of the mean is shown with the error bars. Negative values are indicative of greater dopamine release in the reward versus baseline condition.

The ser9gly SNP was *not* significantly associated with [^11^C]raclopride binding in the ventral striatum or middle caudate at *baseline* (table 2). However, a linear regression analysis controlling for age, sex, diagnosis, and self-reported anhedonia indicated that during receipt of unpredictable monetary reward the glycine allele was associated with a greater reduction in D2/3 receptor binding (i.e. increased reward-related DA release) in the middle caudate (r^2^ explained = 0.24, β-weight = 0.5, t = 3.3, p = 0.003) and the ventral striatum (r^2^ explained = 0.14; β-weight = 0.4, t = 2.3, p = 0.027) (fig. 2, table 2). A similar, but not statistically significant pattern of findings was observed when the MDD group was considered separately from the HC group (figure, S2).

**Table 2:** Linear Regression Showing Effects of Ser9Gly on Raclopride Binding and Anhedonia Scores.

	Predictors	Ser9Gly R^2^ Explained	Beta-Weight	T-Score	P-Value	Durbin-Watson	VIF
Baseline BP_ND_: VS	α	0.043	0.22	1.3	0.202	2.2	1.07
Baseline BP_ND_: MC	α	0.018	0.14	0.9	0.399	2.1	1.07
Δ BP_ND_: VS	α	0.142	0.39	2.3	0.027*	2.0	1.07
Δ BP_ND_: MC	α	0.235	0.50	3.3	0.003**	2.1	1.07

Note: BP_ND_ = Non-Displaceable Binding Potential; Δ BP_ND_ = Change in Raclopride Binding Potential Between Baseline Sensorimotor and Gambling Task; Chapman P = Chapman Physical Anhedonia Rating Scale; Chapman S = Chapman Social Anhedonia Rating Scale; MC = Middle Caudate; SHAPS = Snaith-Hamilton Pleasure Scale; VS = Ventral Striatum.

α = age, sex, diagnosis, SHAPS score, and DRD3 ser9gly (ser/ser vs ser/gly vs gly/gly) in combined sample.

* = p<0.05.

** = p<0.01.

The ser9gly polymorphism was not in HWE (p = 0.0346) most likely because of the small sample size. Although ethnic stratification is also a possible cause of the divergence of the Ser9Gly polymorphism from HWE, [^11^C]raclopride binding was not affected by ethnic origin (table S1) suggesting that our results are not confounded by population admixture.

## Discussion

### A potential neurobiological mechanism of ser9gly DA signaling modulation

Our finding that the glycine allele is associated with greater reward-related DA release is potentially consistent with an *in vitro* study showing that the glycine allele yields D3 autoreceptors that have a higher affinity for DA in both G-protein-coupled and uncoupled receptor states and show more robust cAMP and MAPK signaling [47].

Electrophysiology studies demonstrate that DA neurons in the ventral tegmental area (VTA) are either inactive, display irregular tonic-firing, or show phasic burst-firing patterns. In response to unpredicted reward the population of DA neurons engaged in burst-firing increases, although only those DA neurons that had been active prior to stimulation are able to burst-fire in response to excitatory afferent input [48]. The D3 receptor is densely distributed in the shell region of the NAc where it predominantly acts as a presynaptic autoreceptor that inhibits DA release. The higher affinity of the glycine D3 autoreceptor would be expected to decrease the extrasynaptic DA concentration under conditions of tonic DA release, potentially diminishing the inhibitory tone on the amount of DA released per action potential during burst firing, and thereby enabling an increase in the *efficacy* with which burst-firing releases DA (i.e., more neurotransmitter released per action potential) [49].

The higher affinity of the glycine autoreceptor may also predispose to elevated phasic DA signaling – i.e. greater reward-associated DA release – via a second mechanism. In rodents the tonic firing activity of VTA DA neurons is regulated by antagonistically-acting afferent projections to the NAc from the medial prefrontal cortex (mPFC) and ventral subiculum (vSub) (figure 1). D2/3 receptor stimulation selectively inhibits mPFC inputs to the NAc while D1 receptor agonists increase NAc activity via the vSub [48]. More specifically, excitatory projections from the vSub release inactive DA neurons from inhibition, by stimulating the inhibitory projection from the NAc to the ventral pallidum, thereby increasing the pool of DA neurons available for burst-firing [48]. The D1 and D3 receptors form dimers in the limbic and associative striatum allowing for synergistic interaction [50], such that higher affinity D3 receptors, by augmenting *phasic* DA release, could theoretically potentiate hippocampal drive and thus increase the *population* of DA neurons entering a burst-firing pattern in response to rewarding stimuli. One would therefore expect a D3 receptor with higher affinity for DA to result in greater DA release during phasic burst-firing.

### Potential clinical implications

Depression and/or anhedonia has been shown to be associated with a reduced striatal response to reward [reviewed in [44], [51]]. These data are primarily derived from hemodynamic imaging methods, especially functional MRI (fMRI). The BOLD fMRI signal reflects the corresponding change in regional cerebral blood flow associated with synaptic terminal field metabolic activity and is dominated by glutamatergic transmission, with a relatively smaller component attributable to GABAergic transmission [52]. In contrast, the proportion of the BOLD signal that reflects a direct contribution of terminal field metabolic activity of monoaminergic (i.e., DA) neurons is negligible [52]. Within the striatum many of the DA projections synapse directly on the axon terminals of glutamatergic neurons, and DA release acts to inhibit glutamate release from the afferent excitatory projections [53], [54], Thus an increase in striatal DA signaling could potentially account for the reduction in the BOLD striatal response to reward observed in the ventral striatum in depressed MDD subjects. These data raise the possibility that the glycine allele, which is associated with increased reward-associated DA release, predisposes to the development of mood disorders. Indeed we found that among the MDD patients glycine allele carriers were more anhedonic (measured with the Chapman Social Anhedonia Scale) than serine/serine homozygotes (data not shown). This association between the glycine allele and anhedonia is potentially consistent with preclinical data that indicate that chronic stress and aversive stimulation induce a pronounced activation of the DA system both electrophysiologically (number of DA neurons firing spontaneously) and behaviorally (response to psychostimulants), driven by hyperactivity within the ventral hippocampus [55].

Theoretically, the glycine allele-associated increase in phasic DA signaling may also contribute to the development of addiction disorders. Pharmacological agents that induce feelings of pleasure are known to stimulate DA release in the ventral striatum (reviewed in [56]). In addition, as discussed in Joutsa et al. [57], the binding of [^11^C]raclopride is sensitive to changes in striatal DA concentration during receipt of non-pharmacological rewards such as a video game [58], large monetary wins versus large monetary losses [59] and the monetary incentive delay task [60]. Notably, in a study of pathological gamblers and healthy controls who completed 3 PET scans with [^11^C]raclopride while gambling with a slot machine, the severity of addiction to gambling was positively associated with the degree of DA release in the basal ganglia during gambling [57]. Consistent with these results, highly impulsive individuals, who are thought to be vulnerable to developing addiction disorders, were shown to have diminished availability of striatal D2/3 autoreceptors, potentially predisposing them to a greater phasic DA response [61].

In preclinical models of substance abuse, stress-induced elevations in glucocorticoids result in an increased response to psychostimulants while administration of glucocorticoid antagonists or adrenalectomy attenuates self-administration of psychostimulants [62], [63]. In healthy humans, cortisol levels were found to be positively correlated with amphetamine-induced DA release measured with [^11^C]raclopride in the ventral striatum and the dorsal putamen [64]. These data, taken together with the findings presented here, thus suggest the hypothesis that the susceptibility for developing hyperdopaminergic responses to stress in carriers of the glycine allele may be accentuated in individuals with hypercortisolemia.

### Potential treatment implications

The important role of DA signaling in mood disorders is congruent with the fact that pramipexole, a medication with antidepressant properties that selectively binds to the D3 receptor, has been reported to augment tonic DA release and to alter the sensitivity of D3 autoreceptors in rodents [25]. Interestingly, Liu et al. [65] reported that Parkinson's disease patients homozygous for the serine allele had a 60% response rate to pramipexole compared to only a 13% response rate in glycine allele carriers. The effect of ser9gly on pramipexole response in mood disorders is unknown but merits investigation.

### Additional comments

The ser9gly variant appears to be pleiotropic and is a well established risk factor for movement disorders such as tardive dyskinesia [66], [67] and essential tremor [47], [68]. It has not only been implicated in mood disorders and addiction, but personality traits [69], schizophrenia [70], [71], cognition [72], autism [73], obsessive compulsive disorder [74], and response to clozapine [75], [76] in the context of psychosis. Conceivably, the ser9gly polymorphism is capable of modulating a variety of functions and circuits in which the D3 receptor plays an integral role, perhaps explaining evidence for a recent positive selection sweep in favor of the ser9 allele in European [70] and east Asian populations [71].

The wide range of phenotypes implicated in these genetic association studies together with our results, which are suggestive of a large effect size for the ser9gly variant, raise the question of why the evidence for the D3 receptor involvement in reward and depression-like behavior has not been more robust in the preclinical literature. One possibility is that in contrast to monkeys and primates, which carry the gly9 allele, there is no equivalent polymorphism in mice or rats (figure S3). A D3 receptor knockout may not be adequate to model the change in D3 receptor function associated with the serine-glycine substitution. However, future studies with a gly9 knock-in mouse or rat may provide valuable insight into the role of the D3 receptor in addiction, mood disorders, and psychosis.

One weakness of our study is the modest sample size, which may have led to type I or II error. Although large sample sizes are uncommon in PET studies because of cost and radiation exposure, PET has an advantage over magnetic resonance imaging because it allows a particular molecular target to be assayed directly. Thus it is likely that true signals can be detected with relatively small sample sizes. For example, in a sample of HCs (n = 25) and patients with BD (n = 16) and MDD (n = 17), we previously found a significant association between a non-synonymous polymorphism in the galactose mutarotase gene and serotonin transporter BP in the context of a genome-wide association study [77]. Nevertheless, interactions between ser9gly and functional polymorphisms in DRD2 (C957T and rs1076560), which have been previously associated with D2/3 receptor binding at rest [2], [3], would be interesting to examine in a larger sample.

The order of tasks (sensorimotor and reward task) was not counterbalanced because the PET-[^11^C]raclopride B/I approach permits imaging in only two conditions per scan session and a behavioral reward-induced increase in DA release that displaces [^11^C]raclopride can persist for 45 minutes. Thus the task order was fixed so that the unpredicted reward condition, which we expected to increase phasic DA release, always followed the sensorimotor baseline task. Nevertheless, order effects were unlikely to have affected the assessments of DA release induced by the unpredicted reward task because the [^11^C]raclopride BP obtained during the sensorimotor control task did not differ significantly across genotypes. Future studies potentially may explore the issue of order effects by including a second scan session in which the BP obtained during the sensorimotor control task is compared to the BP obtained during a resting baseline condition (i.e., such a design can address order effects by counterbalancing the order of the session comprised of the resting baseline and sensorimotor task with the session comprised of the sensorimotor task and unpredicted reward task).

Another limitation of our paper concerns the limited temporal resolution of PET, which does not allow a distinction to be made between the effect of the ser9gly DRD3 SNP on DA release associated with anticipation of reward versus DA release associated with receipt of reward.

Future multi-modal imaging studies that combine functional MRI (fMRI) and [^11^C]raclopride PET data may provide further insights into the role of the ser9gly polymorphism in modulating reward-associated DA-release. A number of studies have successfully applied multi-modal imaging in order to examine the relationship between serotonergic function [78], [79] or dopamine storage capacity [80] and the hemodynamic response to affective stimuli in regions such as the amygdala. Conceivably, a gambling task similar to the one used in this study could be implemented in a cohort of subjects who complete both [^11^C]raclopride PET and fMRI. It may also be important to examine the effect of the ser9gly SNP on baseline D3 receptor binding using the selective D3 ligand, [^11^C]-(+)-PHNO.

In summary, the present study demonstrates that the DRD3 ser9gly variant influences *in vivo* DA release in the context of unpredicted reward, a finding with potentially important implications for the pathophysiology and treatment of addiction and mood disorders.

## Supporting Information

Table S1 Linear Regression Showing Effects of Genetically-Defined African and European Ancestry on Raclopride Binding.
(DOC)

Figure S1 **During each trial, subjects were presented four distinct pictures (apple, grape, cherry, bell) presented in a “slot-machine” motif.** (Adapted from Martin-Soelch et al. 2012). Subjects were asked to choose one of the four with a button press on a four-button response box. This response was followed by a 500 msec delay. In the rewarded trials a one-dollar bill appeared for 1,000 msec and subjects heard the sound of an opening cash-register door. These monetary gains were provided in a pseudo-randomized order with an average of one reward every fourth trial. In the sensorimotor control trials, subjects instead were presented with a meaningless symbol accompanied by a clicking sound on every fourth trial. After being made aware of the trial outcome, subjects were presented their running total of earnings for 1,000 msec. Displaying the actual balance account prevented rapid discounting of the rewards presented. At the end of each trial subjects viewed a blank screen for 1,000 msec. During the reward task subjects were unaware of which trial or picture would lead to the receipt of a reward, except that the same picture could not provide a reward in two consecutive trials. Subjects thus were instructed not to select the same picture more than twice in a row.
(TIFF)

Figure S2 **The figure shows the distribution of the percentage change in D2/3 receptor binding stratified according to genotype.** Figure A illustrates the change in D2/3 receptor binding in the middle caudate in the healthy subjects. Figure B shows the change in D2/3 receptor binding in the middle caudate in the MDD subjects. Figure C illustrates the change in D2/3 receptor binding in the ventral striatum in healthy subjects. Figure D shows the change in D2/3 receptor binding in the ventral striatum in MDD patients.
(TIFF)

Figure S3 **The figures illustrate the position of the rs6280 SNP in the DRD3 gene (top) and the conservation among species of the amino acid at residue 9, highlighted within the red box (bottom).** Mice have a serine allele at residue 9, while rats carry a threonine allele. The glycine allele is present in the other species listed in the figure, including humans. The data were obtained from the National Center for Biotechnology Information (www.ncbi.nlm.nih.gov).
(TIF)
