# Supplementary material for: The Functional DRD3 Ser9Gly Polymorphism (rs6280) Is Pleiotropic, Affecting Reward as Well as Movement
Source: PLoS One. 2013 Jan 24;8(1):e54108. doi: 10.1371/journal.pone.0054108 (PMC3554713; doi:10.1371/journal.pone.0054108)
Supplement: Table S1 — Linear Regression Showing Effects of Genetically-Defined African and European Ancestry on Raclopride Binding. (DOC) [file pone.0054108.s001.doc]

**Table S1. Linear Regression Showing Effects of Genetically-Defined African and European Ancestry on Raclopride Binding**

|  | **Predictor** | **R^2^ Explained by Ancestry** | **Beta-Weight** | **T-Score** | **P-Value** |
| --- | --- | --- | --- | --- | --- |
| Baseline BP_ND_: VS | African Ancestry | 0.01 | 0.09 | 0.5 | 0.654 |
| Baseline BP_ND_: VS | European Ancestry | 0.01 | 0.12 | 0.6 | 0.539 |
| Baseline BP_ND_: MC | African Ancestry | < 0.01 | 0.01 | 0.1 | 0.973 |
| Baseline BP_ND_: MC | European Ancestry | 0.03 | 0.16 | 0.9 | 0.403 |
| Δ BP_ND_: VS | African Ancestry | 0.02 | 0.13 | 0.7 | 0.477 |
| Δ BP_ND_: VS | European Ancestry | 0.04 | 0.20 | 1.1 | 0.278 |
| Δ BP_ND_: MC | African Ancestry | 0.01 | 0.12 | 0.5 | 0.619 |
| Δ BP_ND_: MC | European Ancestry | 0.03 | 0.16 | 0.9 | 0.380 |

Note: BP_ND_ = Non-Displaceable Binding Potential; Δ BP_ND_ = Change in Raclopride Binding Potential Between Baseline Sensorimotor and Gambling Task; MC=Middle Caudate; VS=Ventral Striatum.
