# Supplementary figures and images for: The Functional DRD3 Ser9Gly Polymorphism (rs6280) Is Pleiotropic, Affecting Reward as Well as Movement
Source: PLoS One. 2013 Jan 24;8(1):e54108. doi: 10.1371/journal.pone.0054108 (PMC3554713; doi:10.1371/journal.pone.0054108)

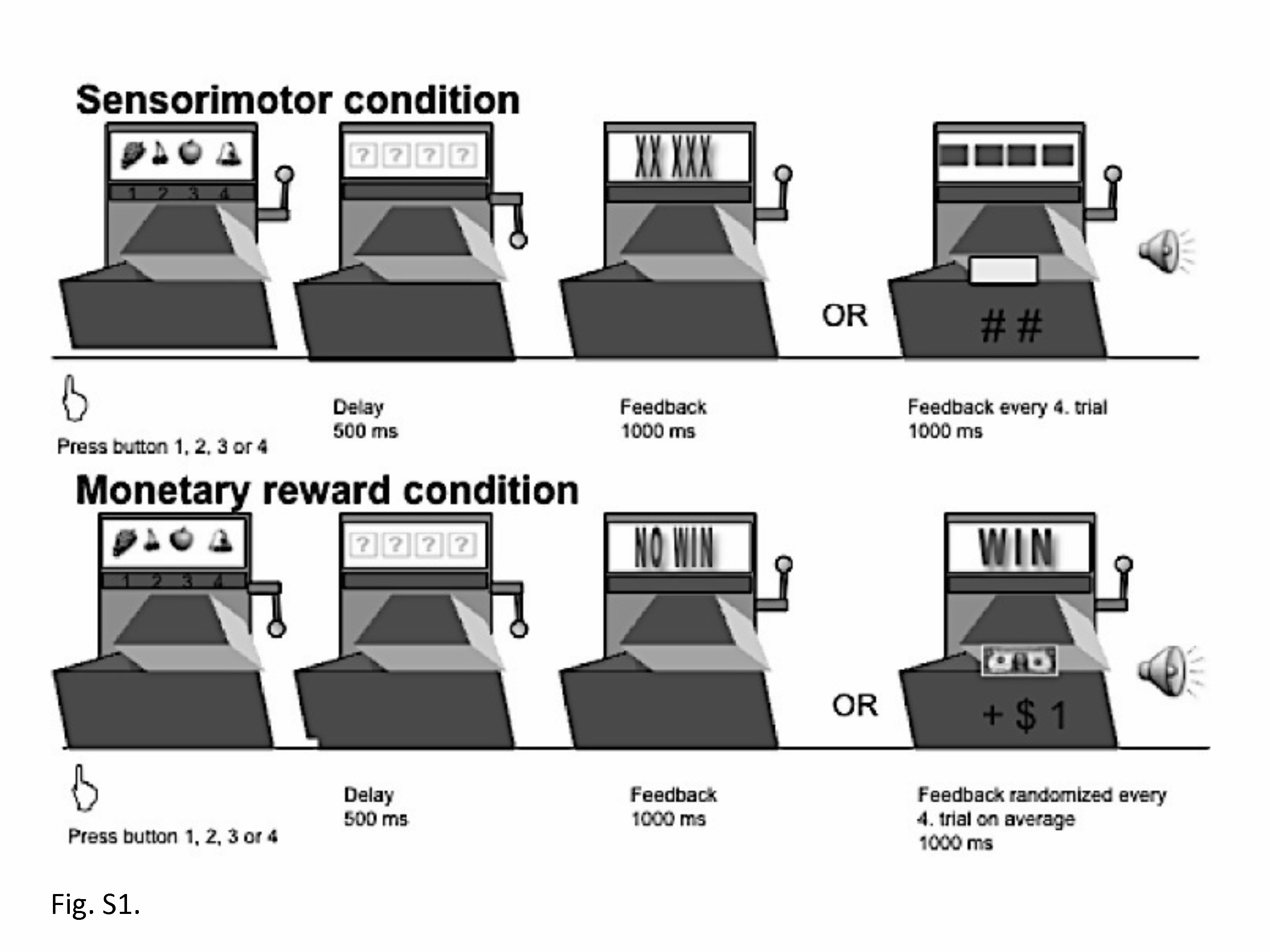

Supplement: Figure S1 — During each trial, subjects were presented four distinct pictures (apple, grape, cherry, bell) presented in a “slot-machine” motif. (Adapted from Martin-Soelch et al. 2012). Subjects were asked to choose one of the four with a button press on a four-button response box. This response was followed by a 500 msec delay. In the rewarded trials a one-dollar bill appeared for 1,000 msec and subjects heard the sound of an opening cash-register door. These monetary gains were provided in a pseudo-randomized order with an average of one reward every fourth trial. In the sensorimotor control trials, subjects instead were presented with a meaningless symbol accompanied by a clicking sound on every fourth trial. After being made aware of the trial outcome, subjects were presented their running total of earnings for 1,000 msec. Displaying the actual balance account prevented rapid discounting of the rewards presented. At the end of each trial subjects viewed a blank screen for 1,000 msec. During the reward task subjects were unaware of which trial or picture would lead to the receipt of a reward, except that the same picture could not provide a reward in two consecutive trials. Subjects thus were instructed not to select the same picture more than twice in a row. (TIFF) [file pone.0054108.s002.tiff]

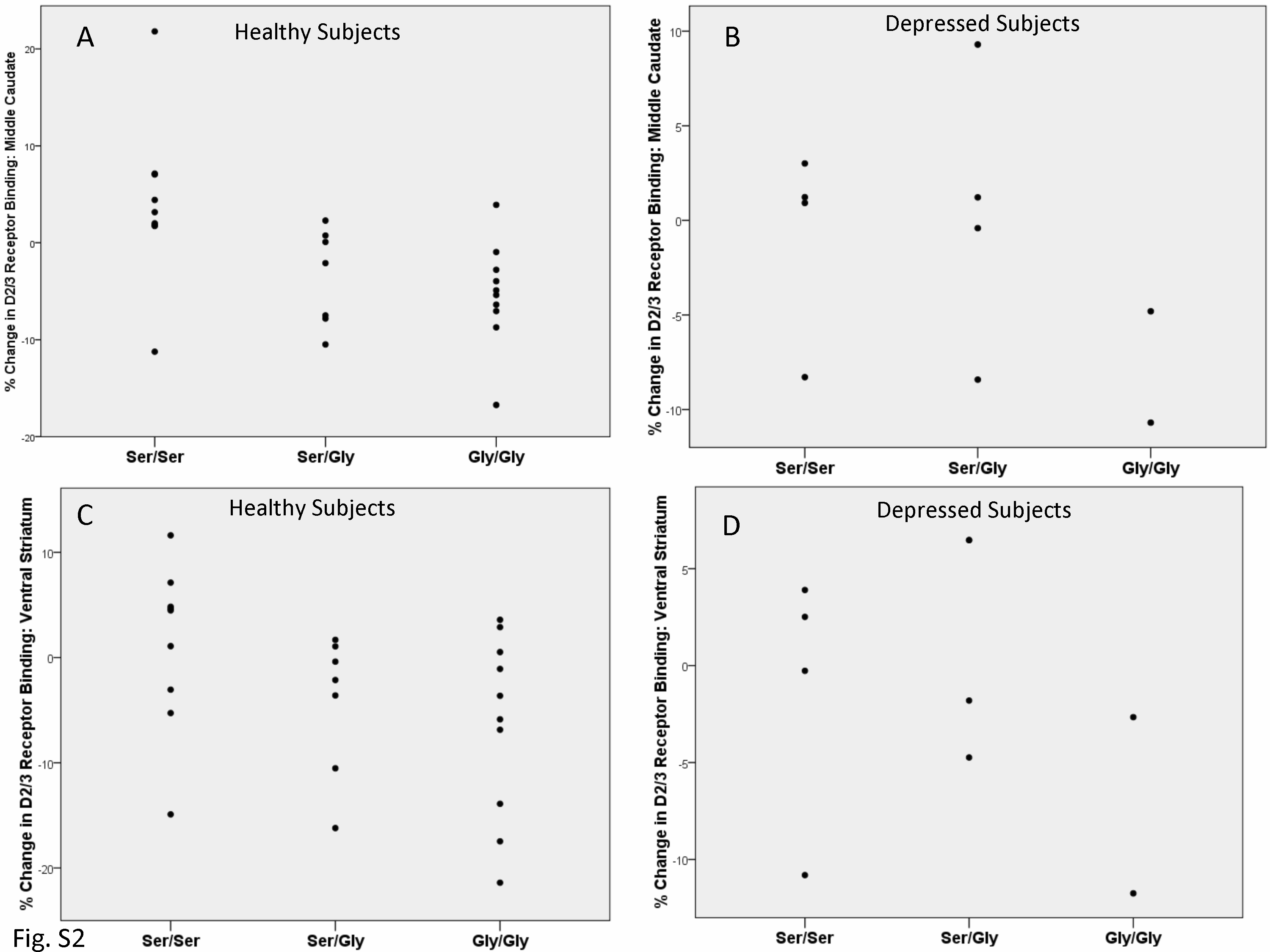

Supplement: Figure S2 — The figure shows the distribution of the percentage change in D2/3 receptor binding stratified according to genotype. Figure A illustrates the change in D2/3 receptor binding in the middle caudate in the healthy subjects. Figure B shows the change in D2/3 receptor binding in the middle caudate in the MDD subjects. Figure C illustrates the change in D2/3 receptor binding in the ventral striatum in healthy subjects. Figure D shows the change in D2/3 receptor binding in the ventral striatum in MDD patients. (TIFF) [file pone.0054108.s003.tiff]

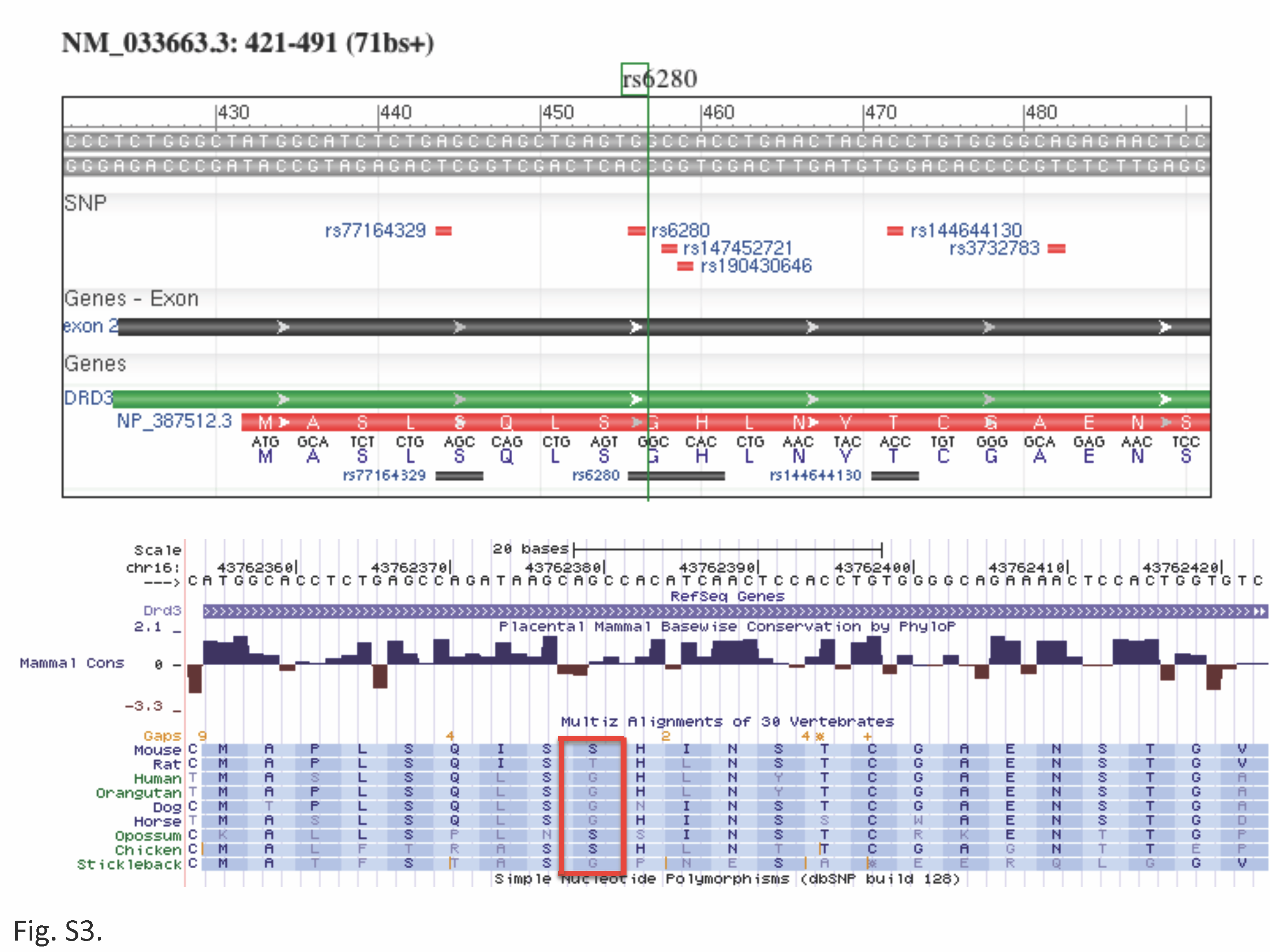

Supplement: Figure S3 — The figures illustrate the position of the rs6280 SNP in the DRD3 gene (top) and the conservation among species of the amino acid at residue 9, highlighted within the red box (bottom). Mice have a serine allele at residue 9, while rats carry a threonine allele. The glycine allele is present in the other species listed in the figure, including humans. The data were obtained from the National Center for Biotechnology Information (www.ncbi.nlm.nih.gov). (TIF) [file pone.0054108.s004.tif]
